# Rumors of disease in the global village: outbreak verification.

**DOI:** 10.3201/eid0602.000201

**Published:** 2000

**Authors:** T W Grein, K B Kamara, G Rodier, A J Plant, P Bovier, M J Ryan, T Ohyama, D L Heymann

**Affiliations:** Department of Communicable Disease Surveillance and Response, World Health Organization, Geneva, Switzerland. greint@who.int

## Abstract

Emerging infectious diseases and the growth of information technology have produced new demands and possibilities for disease surveillance and response. Increasing numbers of outbreak reports must be assessed rapidly so that control efforts can be initiated and unsubstantiated reports can be identified to protect countries from unnecessary economic damage. The World Health Organization has set up a process for timely outbreak verification to convert large amounts of data into accurate information for suitable action. We describe the context and processes of outbreak verification and information dissemination.


### Rumors of Disease in the Global Village: Outbreak Verification

**Thomas W. Grein, Kande-Bure O. Kamara, Guénaël Rodier, Aileen J. Plant, Patrick Bovier, Michael J. Ryan, Takaaki Ohyama, and David L. Heymann**
World Health Organization, Geneva, Switzerland

Emerging infectious diseases and the growth of information technology have produced new demands and possibilities for disease surveillance and response. Increasing numbers of outbreak reports must be assessed rapidly so that control efforts can be initiated and unsubstantiated reports can be identified to protect countries from unnecessary economic damage. The World Health Organization has set up a process for timely outbreak verification to convert large amounts of data into accurate information for suitable action. We describe the context and processes of outbreak verification and information dissemination.

Globalization presents new challenges and opportunities in combating diseases likely to cause epidemics. As a result of increased international travel and trade, local events acquire international importance. At the same time, the rapid global expansion of telecommunications and broadened access to news media and the Internet have changed the way society treats information. Reports of disease outbreaks are more widely disseminated and more easily accessible than ever before. However, the quality of information is no longer controlled and may be provided out of context, often causing unnecessary public anxiety and confusion. Rumors that later prove to be unsubstantiated may lead to inappropriate response, causing disruption in travel and trade and economic loss to affected countries.

The World Health Organization (WHO), speaking for 191 member countries, is uniquely positioned to coordinate infectious disease surveillance and response at the global level. WHO receives reports of disease outbreaks around the world from various sources. While some of these reports are warnings of genuine epidemics, others may reflect endemic disease or may be mere rumors.

To investigate and follow up outbreak reports, WHO established an innovative mechanism—outbreak verification—in early 1997. Outbreak verification is a new approach to global disease surveillance (1). Its aim is to improve epidemic disease control by informing key public health professionals about confirmed and unconfirmed outbreaks of international public health importance.

Address for correspondence: Thomas Grein, Department of Communicable Disease Surveillance and Response, World Health Organization, 20 Avenue Appia, CH-1211 Geneva 27, Switzerland; fax: 41-22-791-4198; e-mail: greint@who.int.

### The Outbreak Verification System

The outbreak verification system follows the general principles of surveillance: systematic collection, collation, analysis, and interpretation of data and dissemination to those who need the information for action (Figure 1). Data derived from an extensive network of information sources are transformed by the outbreak verification team into timely, accurate information about important disease outbreaks.

When the outbreak verification team receives an unconfirmed outbreak report, the relevance to international public health is assessed, and, if appropriate, further information is sought. Once an outbreak is substantiated and considered of public health importance, information is rapidly disseminated to a network of international partners.

### Sources of Information

Outbreak verification is based on a broad range of information sources, including national institutes of public health, WHO offices at regional and national level, the United Nations

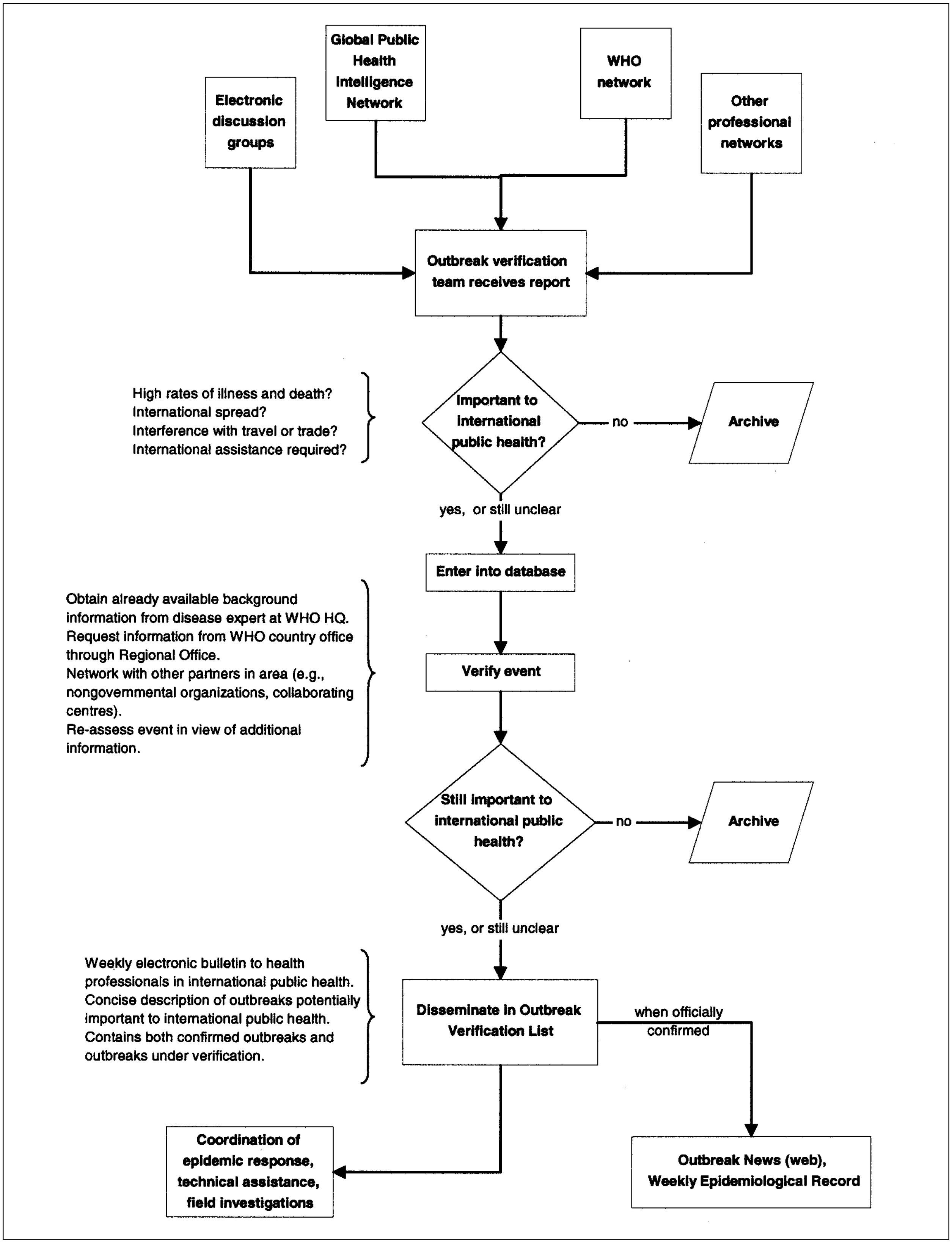

Figure 1. Outbreak verification at the World Health Organization.

system, nongovernmental organizations, WHO collaborating centers, newspapers, television, and radio (2). With the advent of modern communication technologies, many initial outbreak reports now originate in the electronic media and electronic discussion groups. Indeed, the abundance of outbreak-related documents on the World Wide Web presents a challenge: identifying reports of global public health importance.

The tasks of identifying and extracting outbreak reports from the electronic media is mainly performed by the Global Public Health Information Network (GPHIN), an electronic surveillance system developed by Health Canada. GPHIN continuously monitors some 600 sources, including all major news wires, newspapers, and biomedical journals. The system focuses its search on communicable diseases but will soon also cover noncommunicable diseases, food and water safety, environmental health risks, and the health impact of natural disasters (3). The quality of reports retrieved by GPHIN varies considerably, and information may be presented out of context (4).

Other information providers are the Internet and electronic-mail-based discussion groups. Their scope and readership may be worldwide (e.g., ProMed), regional (e.g., PACNET in the Pacific region), or specific (e.g., TravelMed). These groups can be accessed through free and unrestricted subscription. Because they receive outbreak information from many sources, including sources other than the electronic media, they are valuable information providers (5).

### Selection of Outbreak Reports for Verification

The verification team first determines if an event is of potential international public health importance. International public health importance has been defined as serious health impact or unexpectedly high rates of illness and death; potential for spread beyond national borders; interference with international travel or trade; or likely need for international assistance in disease control.

Each event is assessed individually on the basis of these criteria. While some diseases will almost always be regarded important for international public health (e.g., Ebola hemorrhagic fever, cholera), others may not, depending on the circumstances.

### Process of Verification

Once an event has been assessed as of potential international importance, the process of verification is initiated.

The outbreak verification team establishes the potential importance of the event, on the basis of available background information, endemicity levels, and details of previous outbreaks. This information is then shared by e-mail with designated contacts in WHO regional offices, who seek confirmation of details from health authorities in the countries concerned, usually through the WHO representative. The outbreak verification team may seek additional information from other organizations in the field, such as the International Red Cross, Médecins sans Frontières, and Medical Emergency Relief International.

Upon receipt of feedback, the outbreak verification team determines if the event meets the definition of an outbreak (observed number of cases exceeds expected number of cases in a given population for a given period) and the criteria for international public health importance. Reaching a final decision may require further consultation with the WHO regional office or the country representative or health authorities in-country.

### Dissemination of Information

Timely dissemination of outbreak information to those who need to know is a key aspect of the outbreak verification process, and details of outbreaks with potential for international public health importance are disseminated through various channels. Information is shared directly with partners for immediate action (epidemic response) but also routinely with a wider audience through the Outbreak Verification List, the WHO Disease Outbreak News on the World Wide Web, and the Weekly Epidemiological Record (WER).

The Outbreak Verification List is distributed weekly by e-mail to approximately 800 subscribers. The distribution list includes WHO staff worldwide, other UN agencies, national health authorities, field epidemiology training programs, and nongovernmental organizations. Because the Outbreak Verification List is not an official WHO publication, its distribution is limited to subscribers.

The WHO Disease Outbreak News is on the WHO web page and provides the public with information about outbreaks of international importance. Often events that initially appeared in the Outbreak Verification List are subsequently reported in Outbreak News. Because Outbreak News is in the public domain, only information about officially confirmed outbreaks is disseminated. Outbreak News (http://www.who.int/emc/outbreak_news/index.html) is one of the most frequently accessed sites on the WHO home page.

The third mechanism for communicating outbreak-related information is the WER. This report is published in French and English and issued in print and electronically (http://www.who.int/wer/index.html). It covers epidemiologic information on cases and outbreaks of diseases under the International Health Regulations (yellow fever, plague, cholera) and also on other communicable diseases of public health importance. Recently, an Outbreak News section mirroring the Outbreak News on the web page has been added to the WER.

### Outbreak Response

Coordination of timely and effective epidemic response is intrinsically linked to dissemination of information about important disease outbreaks. During the verification process, WHO routinely offers technical assistance for the investigation and control of the event. Such assistance may range from advice (e.g., identifying appropriate laboratory facilities) to deployment of field teams. WHO coordinates the deployment of field teams, drawing from within WHO and among collaborating centers and other international partners.

### Effectiveness of Outbreak Verification

From July 1, 1997, to July 1, 1999, the outbreak verification team identified 246 outbreak reports of potential importance for world health and disseminated them in the Outbreak Verification List. Of the 246 outbreaks, 43% occurred in the African region of WHO; 12% each in the regions of the Americas, eastern Mediterranean, and Europe; 11% in the Southeast Asian region; and 9% in the Western Pacific region. Countries subject to complex emergencies were involved in 121 (49%) outbreaks and industrialized countries in 6 (2%) events.

The most common diseases or syndromes disseminated in the Outbreak Verification List were cholera (n = 78), acute hemorrhagic fevers (n = 24), and acute diarrheal diseases (n = 22). In two (0.8%) cases, the Outbreak Verification List disseminated information about events that could not be substantiated later (Figure 2). Seventy-one percent of the initial reports were retrieved from informal or unofficial sources (e.g., the media, electronic discussion groups, nongovernmental organizations), and 29% were provided by official sources (e.g., WHO network, Ministries of Health). Unofficial sources were the

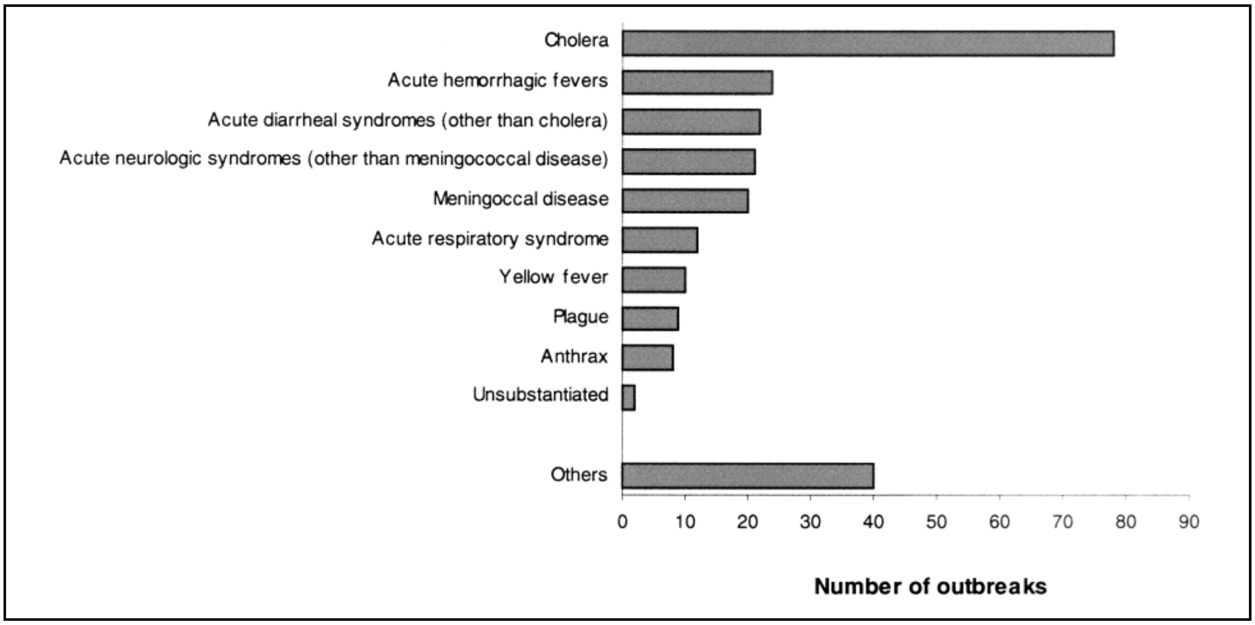

Figure 2. Reports of outbreaks disseminated in Outbreak Verification List, July 1, 1997, to July 1, 1999 (n = 246).

most frequent providers of initial information in all WHO regions and for all diseases, including those subject to the International Health Regulations (cholera, plague, yellow fever).

Information about the date of onset of an outbreak was available in 134 (55%) cases. The median time between reported onset of an outbreak and the outbreak verification team's receipt of the first report was 18 days (from 1 to 215 days). This interval was similar for official and unofficial sources but varied considerably for different diseases: 13 to 15 days (median) for acute hemorrhagic fevers, anthrax, and cholera; 20 to 35 days (median) for yellow fever and plague; and >50 days (median) for acute respiratory syndrome and meningococcal disease. Most reports were verified within a few days and important events usually within <48 hours. The median time between receipt of a first report and appearance of the event in the weekly Outbreak Verification List was 3 days (0 to 69 days).

In addition to the 246 disseminated outbreak reports, 69 events were verified from July 1, 1997, to July 1, 1999, but were not reported in the Outbreak Verification List. Follow up was undertaken because initial reports suggested international public health importance. Of the 69 events, 58 (84%) were excluded from the Outbreak Verification List because they did not meet the criteria for outbreaks or for international public health importance. Four (6%) reports were unsubstantiated, including two reports of smallpox, one of yellow fever, and one of viral hemorrhagic fever. In seven (10%) events, follow up could not be completed, and the verification process remained inconclusive. The 69 excluded events did not differ from the 246 disseminated outbreaks with regard to their distribution by WHO region, initial source of information, or type of disease or syndrome. A reassessment of the 62 verified events did not identify any outbreaks that should have been classified retrospectively as of international importance.

Whenever the outbreak verification team invokes a verification process, assistance to the country in which the event takes place is offered directly by WHO headquarters or through the WHO regional and country offices. Past examples of such assistance include supply of essential materials to outbreak sites, transport of laboratory specimens from the field to appropriate diagnostic facilities, organization of vaccination programs, training of field staff as part of outbreak control measures, or deployment of field teams for disease control. Recent examples of direct assistance by WHO and its partners in field investigations include support for Rift Valley fever in Kenya and Somalia (6), monkeypox in the Democratic Republic of the Congo (7), avian influenza (H5N1) in Hong Kong, Special Administrative Region of China, Ebola hemorrhagic fever in Gabon (8), relapsing fever and acute respiratory infections in southern Sudan, influenza in Afghanistan, and Marburg virus infection in the Democratic Republic of the Congo.

### Conclusions

Outbreak verification is a new approach to global disease surveillance. Its aim is to improve epidemic disease control by providing accurate and timely information about important disease outbreaks. While the outbreak verification concept has remained unchanged since its start in early 1997, its daily application continues to evolve as more data are gathered and more experience is gained.

Currently, most outbreak reports are received from the media, and field personnel are mainly contacted for assistance with verifying reported events. This approach is subject to information bias, which results from the uneven dispersal and use of modern technology throughout the world. Also, different languages are not equally represented in the news media or addressed by electronic search engines. While these shortcomings are partly offset by the information received directly from the WHO network, a more active dialogue should be established with field personnel. Receiving primary information directly from the field will lead to earlier detection of important events and events that escape identification. Although thought to be small, the number of important outbreaks recognized only locally is unknown.

The number of outbreak reports selected for verification is small compared with the number of reports received by the outbreak verification team. While the criteria for selecting outbreak reports for verification have been established, their application requires an individual assessment of each single event. Some see in this selection process a lack of transparency and argue that the reader is the best judge of what to believe. This may be the case for those who have time, good information networks, and access to advanced communication technology. However, most international public health workers have none of these and are poorly informed about such events. WHO therefore considers that sharing filtered information is valuable. In a recent survey among the Outbreak Verification List recipients, 72% percent of the respondents stated that the list was very useful or indispensable to their work, and 70% cited the list as their first source of information about a particular event.

Applying the selection criteria is also difficult if available information is insufficient to determine if an event should be classified as an outbreak (number of cases in excess of expected numbers). This problem arises particularly when dealing with endemic diseases in the absence of established epidemic thresholds. The Outbreak Verification List addresses the issue by mentioning events with clear implications for international public health that are not regarded as outbreaks in a separate Notes section. The Outbreak Verification List shares relevant and often sensitive information with public health professionals while the verification process is still under way. Although this has led on rare occasions (<1%) to the dissemination of information about unsubstantiated events, the Outbreak Verification List usually provides timely and accurate information about important disease outbreaks.

Because of its confidential nature, the Outbreak Verification List is not in the public domain, and some argue that WHO is not timely in addressing the information needs of the public about epidemics (4). However, WHO communicates information as soon as it is verified. In some instances, this takes time, but the delay prevents release of inaccurate information.

Industrialized countries feature infrequently in the Outbreak Verification List because it is assumed that they can deal with outbreak situations. This is, of course, not always true and leads to an overrepresentation of developing countries in the Outbreak Verification List. However, most outbreaks in developing countries are contributed by nations with complex emergencies. While the reporting may accurately reflect the breakdown of the public health and social infrastructures, it may also contain an element of overreporting due to heightened media attention associated with complex emergencies.

As a new concept, early outbreak verification efforts focused mainly on the development of process indicators (information gathering, verification, information dissemination). More outcome-oriented indicators need to be addressed to assess the outbreak verification impact at country level and within WHO. While providing public health professionals with timely and accurate information about important disease outbreaks improves epidemic preparedness and response, this has not been quantified. Possible outcome indicators could include the time interval between first report and the commencement of investigation and control efforts or the proportion of outbreaks with laboratory confirmation. Additional tasks to be addressed in the future are more detailed analyses, including electronic and print mapping to provide both baseline (endemic) and outbreak information, and standardized reports to regions and countries.

Dr. Grein is a medical officer in the Department of Communicable Disease Surveillance and Response at the World Health Organization in Geneva, Switzerland. His activities at WHO include the investigation and control of epidemics and training in field epidemiology.

### References

1. Heymann DL, Rodier GR. Global surveillance of communicable diseases. Emerg Infect Dis 1998;4:362-5.
2. World Health Organization. Summary records and reports of committees of the 48th World Health Assembly. Geneva: the Organization; 1995 Report No. WHA48/1995/REC/3.
3. Cripp R. Docs using net as disease detector. Wired News [serial online] 1998 Apr. Available from URL: http://www.wired.com/news/news/technology/story/11466.htm
4. Eysenbach G, Diepgen TL. Towards quality management of medical information on the internet: evaluation, labelling, and filtering of information. BMJ 1998;317:1496-502.
5. Taubes G. Virus hunting on the web. Technology review [serial online] 1998 Nov-Dec. Available from URL: http://www.techreview.com/articles/nov98/taubes.htm
6. An outbreak of Rift Valley fever, Eastern Africa, 1997-98. Wkly Epidemiol Rec 1998;73:105-12.
7. Human monkeypox in Kasai Oriental, Democratic Republic of the Congo (former Zaire)-preliminary report of October 1997 investigation. Wkly Epidemiol Rec 1997;72:365-72.
8. Ebola haemorrhagic fever. A summary of the outbreak in Gabon. Wkly Epidemiol Rec 1997;72:7-8.
